# Supplementary material for: Splice-Junction-Based Mapping of Alternative Isoforms in the Human Proteome
Source: Cell Rep. Author manuscript; Available in PMC 2020 Jan 15. (PMC6961840; doi:10.1016/j.celrep.2019.11.026)
Supplement: 2 [file NIHMS1546469-supplement-2.zip › DF1/PXD006675/LeftAtrium/LeftAtrium_41_PLCD1_VGVPLAGEEWDAR.pdf]

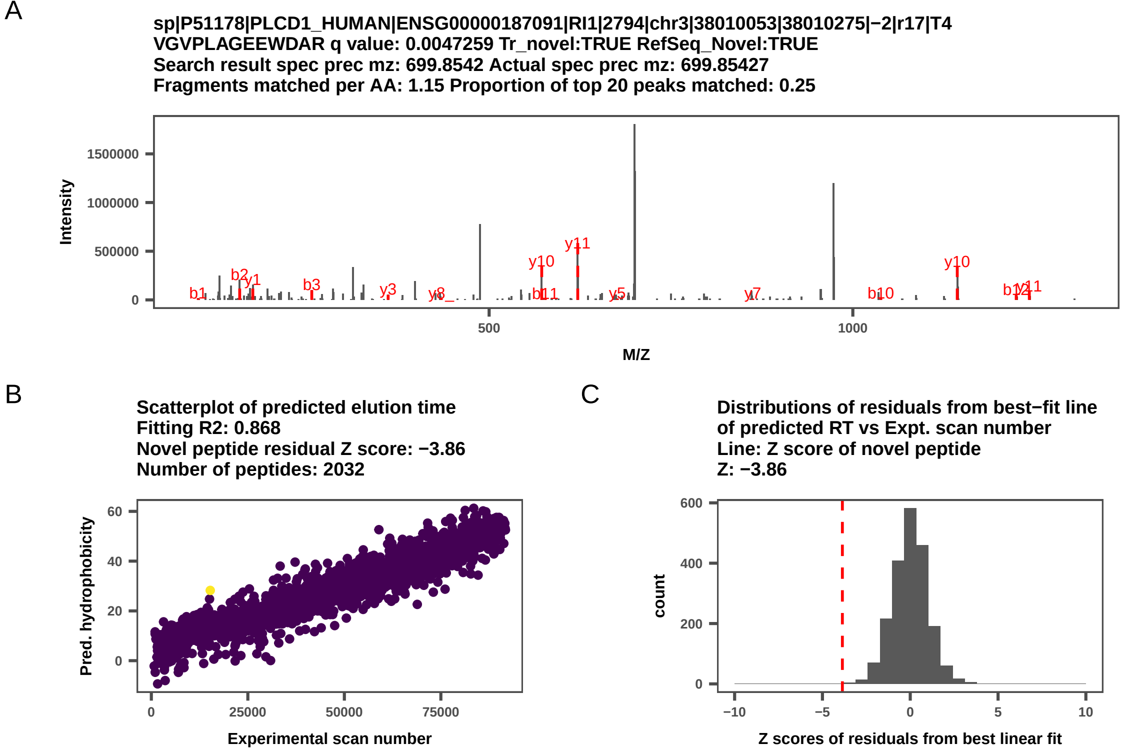
A
sp|P51178|PLCD1_HUMAN|ENSG00000187091|RI1|2794|chr3|38010053|38010275|−2|r17|T4
VGVPLAGEEWDAR q value: 0.0047259 Tr_novel:TRUE RefSeq_Novel:TRUE
Search result spec prec mz: 699.8542 Actual spec prec mz: 699.85427
Fragments matched per AA: 1.15 Proportion of top 20 peaks matched: 0.25
Intensity
1500000
1000000
500000
0
M/Z
500
1000
b1
b2
y1
b3
y3
y8
y10
b11
y11
y5
y7
b10
y10
b12
y11
B
Scatterplot of predicted elution time
Fitting R2: 0.868
Novel peptide residual Z score: −3.86
Number of peptides: 2032
Pred. hydrophobicity
60
40
20
0
0
25000
50000
75000
Experimental scan number
C
Distributions of residuals from best−fit line
of predicted RT vs Expt. scan number
Line: Z score of novel peptide
Z: −3.86
count
600
400
200
0
−10
−5
0
5
10
Z scores of residuals from best linear fit
